# Gradient-Enhanced Modelling of Damage for Rate-Dependent Material Behaviour—A Parameter Identification Framework

**DOI:** 10.3390/ma13143156

**Published:** 2020-07-15

**Authors:** Robin Schulte, Richard Ostwald, Andreas Menzel

**Affiliations:** 1 Institute of Mechanics, TU Dortmund University, Leonhard-Euler-Str. 5, 44227 Dortmund, Germany; robin.schulte@udo.edu (R.S.); richard.ostwald@udo.edu (R.O.); 2 Division of Solid Mechanics, Lund University, P.O. Box 118, 22100 Lund, Sweden

**Keywords:** rate-dependent material behaviour, gradient-enhanced damage at large strains, parameter identification, finite elements

## Abstract

The simulation of complex engineering components and structures under loads requires the formulation and adequate calibration of appropriate material models. This work introduces an optimisation-based scheme for the calibration of viscoelastic material models that are coupled to gradient-enhanced damage in a finite strain setting. The parameter identification scheme is applied to a self-diagnostic poly(dimethylsiloxane) (PDMS) elastomer, where so-called mechanophore units are incorporated within the polymeric microstructure. The present contribution, however, focuses on the purely mechanical response of the material, combining experiments with homogeneous and inhomogeneous states of deformation. In effect, the results provided lay the groundwork for a future extension of the proposed parameter identification framework, where additional field-data provided by the self-diagnostic capabilities can be incorporated into the optimisation scheme.

## 1. Introduction

The adequate numerical prediction of material behaviour under complex loads and boundary conditions is of high significance in the product development context, particularly in view of today’s trends towards more efficient and cost-effective production, but also in view of component safety requirements and appropriate component lifetime predictions. To this end, not only the material model formulation itself needs to be physically sound and able to capture all relevant material phenomena, but the corresponding model parameters also need to be identified properly. This process of model calibration is typically carried out with the help of optimisation-based parameter identification schemes.

In this work, we present an optimisation-based framework that enables the identification of parameters for rate-dependent materials under large strains and subject to gradient-enhanced damage by using an efficient two-step approach. In the first step, basic constitutive parameters, in particular elastic parameters, are calibrated based on experiments reflecting homogeneous states of deformation. These parameters are then kept fixed in view of the second step of model calibration, which uses an optimisation scheme based on inhomogeneous states of deformation. The inhomogeneous states of deformation activate gradient terms within the gradient-enhanced damage formulation considered in this work, so that mesh-objective damage effects can be captured within finite element-based simulations. With damage gradient terms activated due to inhomogeneous states of deformation, we proceed to the identification of damage-related model parameters in the second step of the model calibration procedure.

The particular material considered in this work is an elastomer with self-diagnostic capabilities. Self-diagnostic poly(dimethylsiloxane) (PDMS) elastomers are produced by the addition of so-called mechanophore units, which generate a chemical response towards mechanical loads, as discussed by, e.g., Brighenti and Artoni [1]. The specific PDMS elastomer containing a supramolecular detection probe was presented and developed by Früh et al. [2]. In this case, the elastomer reacts with strain-dependent levels of fluorescence when illuminated with UV light, thus enabling an in situ quantification of the material’s strain and, potentially, damage state. In this work, however, we restrict our elaborations to the purely mechanical properties of the elastomer, where solely the inhomogeneous displacement field and the load-deflection curve are considered within the model calibration process. A possible future extension of the framework proposed in this work could include an additional consideration of the fluorescence field emitted by the self-diagnostic specimen within the optimisation-based parameter identification procedure.

Different material models allow for the description of the mechanical behaviour of rate-dependent—or rather viscoelastic—materials such as the elastomer considered, mostly using either a convolution integral approach or a differential operator form of the constitutive relation. Reese and Govindjee [3], for example, discussed a non-linear viscoelastic model that utilises a non-linear evolution law that is valid for arbitrary deviations from thermodynamic equilibrium. Bergström and Boyce [4] proposed a constitutive model for the time-dependent behaviour of elastomers, motivated micromechanically by the relaxation of a single entangled chain in a polymer gel. The constitutive viscoelasticity model used in this work is based on a convolution integral approach. The model is based on the well-established finite hyperelasticity framework with uncoupled volumetric-isochoric response that was extended towards viscous effects by Simo and Hughes [5]. The coupling of finite viscoelasticity with gradient-enhanced damage is carried out within the damage regularisation framework provided by Ostwald et al. [6]. This framework is in line with the works of Forest [7] and Miehe [8]. Kiefer et al. [9] presented another approach for a coupling of gradient damage with inelasticity, and Sprave et al. [10] computed complex boundary value problems based on a coupled gradient-enhanced damage formulation. For further, advanced concepts in the context of gradient damage, see, e.g., the work by Liu and Jeffers [11] and the references cited therein.

A corresponding parameter identification scheme for finite viscoelastic material models was introduced by Kleuter et al. [12], where an error square functional—comparing experimentally-obtained inhomogeneous displacement fields with related finite element-based simulations—is minimised by using a gradient scheme. This gradient-based calibration process for finite viscoelasticity was studied in more detail and applied to different materials in [13]. The identification of material parameters for inelastic materials, using experimental data representing non-uniform stress and displacement distributions from the surface of the three-dimensional specimen, was established in the work of Mahnken and Stein [14]. An application of a parameter identification scheme for different constitutive models was presented in [15] where different identification methods, instabilities in least squares problems, and identification for small and non-uniform finite deformations were discussed. Hartmann [16] estimated the constitutive constants of hyperelasticity relations of the generalised polynomial-type and discussed the aspects related to the gradient and convexity behaviour in certain deformations. The particular optimisation methods that are generally applicable were illustrated in, e.g., [17,18,19,20,21].

The present paper is organised as follows: Section 2 introduces the experimental data used in this work, both for homogeneous deformations and for inhomogeneous deformations, as provided in Section 2.1 and Section 2.2, respectively. In Section 3, the constitutive framework is introduced, starting with a brief review of the uncoupled finite hyperelasticity framework in Section 3.1. The extension towards viscous effects is provided in Section 3.2 before the coupling to gradient-enhanced continuum damage is established in Section 3.3. The parameter identification framework used, as well as the results obtained are described in detail in Section 4 before the paper is concluded in Section 5.

## 2. Experiments

**Figure 1:**
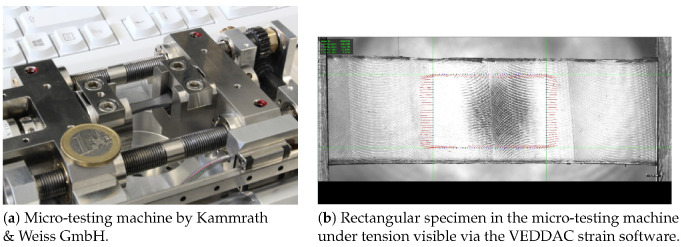
Experimental setting—the micro-testing machine by Kammrath & Weiss GmbH in combination with the CCD camera system by Chemnitzer Werkstoffmechanik GmbH providing load-displacement data, as well as the displacement field on the surface.

In the following, all accomplished types of experiments for the self-diagnostic PDMS elastomer are briefly presented in order to provide an overview of the experimental data required for the subsequent parameter identification of the implemented material model. For most of the experiments, a micro-testing machine by Kammrath & Weiss GmbH (Dortmund, Germany) was used; cf. Figure 1a. The machine can be combined with a CCD camera system from Chemnitzer Werkstoffmechanik GmbH (Chemnitz, Germany), which is of importance considering the need to track the displacement field for the inhomogeneous deformation tests. Figure 1b presents a screenshot of the software VEDDAC strain. The software was linked with the CCD camera and provided additional information on the displacement field during the experiment. The CCD camera additionally took a photo during each time step and delivered the changes in the displacement field directly to the DDS3 software of the testing machine. In the post-processing, the software VEDDAC could be used to extract all required displacement information from the photos taken during the experiment. Since the material was a transparent soft polymer, a stochastic pattern was sprayed onto the specimens with graphite spray. Consequently, the software was able to track all specific positions of the specimen up to the end of the experiment. The following experiments were conducted with virgin material samples, thereby avoiding the influences of Mullin’s effect or the strain history on the material response. The environmental conditions were room conditions, including room temperature.

### 2.1. Homogeneous Deformation Tests

**Figure 2:**
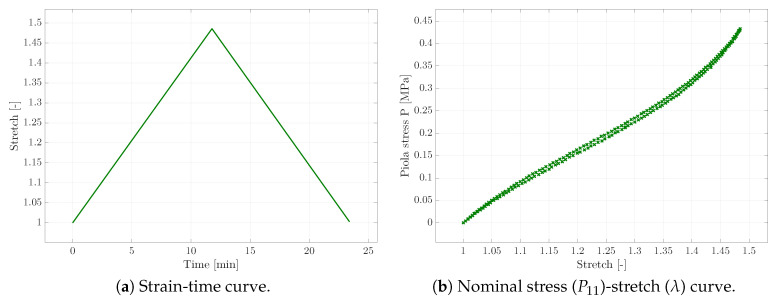
Tensile test results of the micro-testing machine, where the strain-rate is applied (**a**) in order to get the nominal stress (**b**).

**Figure 3:**
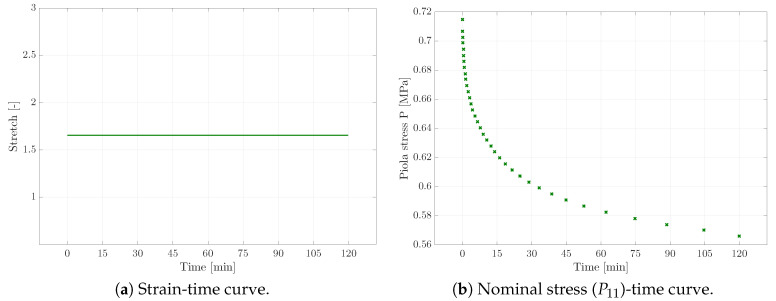
Relaxation test results of the micro-testing machine, where the stretch level is fixed (**a**) in order to get the nominal stress decrease over time (**b**).

**Figure 4:**
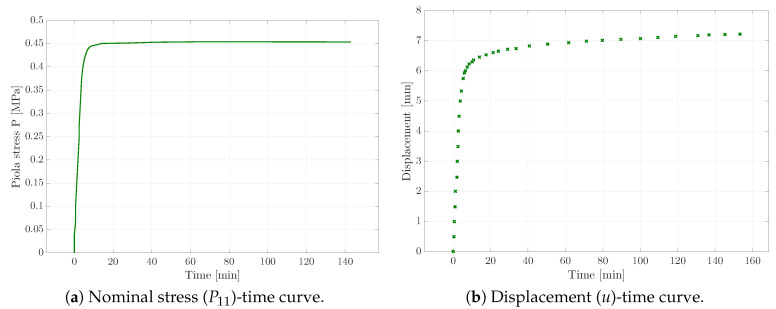
Creep test results of the micro-testing machine, where the stress is fixed over time (**a**) in order to measure the displacement change (**b**).

As a first step, experiments with homogeneous deformation states were conducted. In order to receive sufficient experimental data for the calibration of the viscoelastic material parameters, three different types of experiments were carried out. Apart from uni-axial tensile tests (cf. Figure 2), relaxation tests (see Figure 3), as well as creep tests (cf. Figure 4) provided the experimental data basis for the following parameter identification; see Section 4. Tensile tests with different strain rates were not considered here, as we instead focused on capturing the viscosity-related parameters of the material via the explicit incorporation of creep and relaxation characteristics within the error square functional. In the case of all these experiments, a homogeneous rectangular specimen with an initial length of 25mm, depth of 2.2mm, and width of 10mm was used. An example of the specimen is shown in Figure 1b at the beginning of the corresponding loading path.

### 2.2. Inhomogeneous Deformation Tests

**Figure 5:**
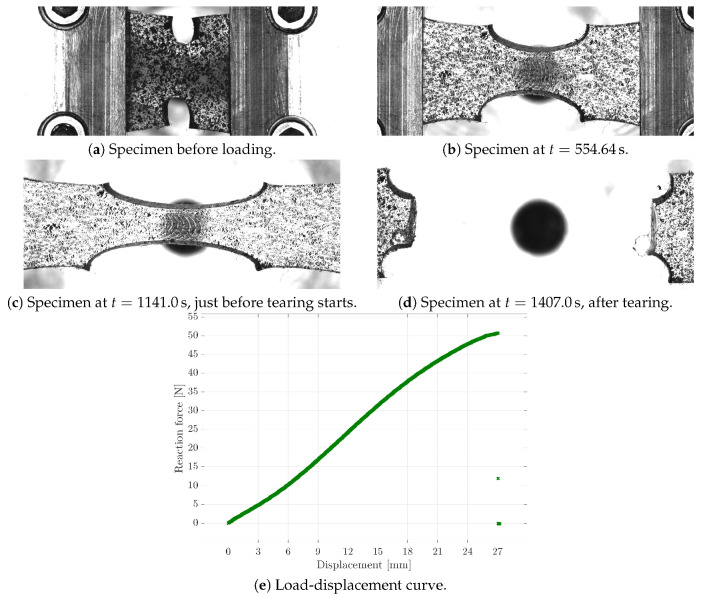
Experimental results of the tensile test of a notched specimen presenting photos of the sample at characteristic time steps, as well as the load-displacement curve.

Apart from the experiments with homogeneous deformation states, an additional experiment with an inhomogeneous deformation state was carried out in order to identify the damage-related material parameters. Considering the regularised gradient-enhanced damage framework, specific information on localisation effects was needed for the identification. Hence, with regard to the possible specimen dimensions, a notched rectangular shape was chosen; see Figure 5a. A constant displacement rate of 20.0μm/s was applied until the sample with an initial length of 10mm, width of 10mm, radius of 2mm, and thickness of 2.2 mm tore apart. In the figure, prominent states of the experiment are visualised. Moreover, Figure 5e shows the corresponding load-displacement curve.

## 3. Modelling Framework—Finite-Strain Viscoelasticity Coupled with Gradient-Enhanced Continuum Damage

Viscoelastic material models can describe a large class of history- and rate-dependent materials. These materials differ from ideally elastic materials by time-dependent effects such as creep or relaxation. Polymeric rubber materials such as the elastomer considered in this work are typical representatives of this class of materials. Constitutive models for viscoelasticity either use a differential operator or convolution integral representation, where the constitutive model considered in this work is based on the latter approach. The convolution integral approach was described in detail in the works by Simo and Hughes [5], Kaliske and Rothert [22], amongst others. The model formulation considered in this work is introduced as follows: Section 3.1 briefly summarises the classic finite strain hyperelastic constitutive relations with volumetric-isochoric decomposition. Section 3.2 proceeds with the generalisation of these relations to a finite strain viscoelastic model. An extension towards gradient-enhanced continuum damage is then briefly covered in Section 3.3.

### 3.1. Finite Hyperelasticity with Volumetric-Isochoric Decomposition

This section briefly outlines the hyperelastic local constitutive setting considered in the work at hand, which will then be extended towards finite viscoelasticity. The constitutive framework at hand facilitates the accommodation of any arbitrary hyperelastic response based on a local Helmholtz free energy function $\psi_{\mathrm{loc}}$, enabling the use of higher order constitutive relations such as the Yeoh model.

Let $F=\nabla_{\;X}\varphi \left(X,t\right)$ denote the deformation gradient, where $x=\varphi \left(X,t\right)\in B_{t}$ reflects the current placement of material points with position $X\in B_{0}$ in the referential placement. The isochoric, i.e., volume-preserving, part $\bar{F}$ of the deformation gradient F is defined as

(1) $$\bar{F}:=J^{-\frac{1}{3}}F\;,\;\mathrm{with}\;J=\det\left(F\right)\;\mathrm{and}\;\det\left(\bar{F}\right)=1\;.$$

In this context, $J^{\frac{1}{3}}\;I$ is referred to as the volumetric part of the deformation gradient, with I denoting the second-order identity tensor. The standard and isochoric right Cauchy–Green deformation tensors are introduced via

(2) $$C:=F^{t}\cdot F\;\mathrm{and}\;\bar{C}:=\bar{F}{}^{t}\cdot \bar{F}\;=J^{-\frac{2}{3}}\;C,$$

respectively.

In view of the hyperelastic contribution to the constitutive response, a local Helmholtz free energy function $\psi_{\mathrm{hyp}}$ of the form

(3) $$\psi_{\mathrm{hyp}}\left(C\right)=\psi_{\mathrm{vol}}\left(J\right)+\psi_{\mathrm{ich}}\left(\bar{C}\right)$$

is considered, where an additive decomposition into volumetric and isochoric energy contributions, $\psi_{\mathrm{vol}}$ and $\psi_{\mathrm{ich}}$, respectively, is taken into account.

Within this modelling framework, the hyperelastic part of the Piola–Kirchhoff stress tensor follows as

(4) $$S_{\mathrm{hyp}}:=2\;\partial_{C}\psi_{\mathrm{hyp}}\left(C\right)=J\;\partial_{J}\psi_{\mathrm{vol}}\left(J\right)\;C^{-1}+{\bar{S}}_{\mathrm{hyp}}\;,$$

where the isochoric contribution to the hyperelastic part of the Piola–Kirchhoff stress tensor is abbreviated as

(5) $${\bar{S}}_{\mathrm{hyp}}:=J^{-\frac{2}{3}}\left[2\;\partial_{\bar{C}}\psi_{\mathrm{ich}}\left(\bar{C}\right)-\frac{1}{3}\;\left[2\;\partial_{\bar{C}}\psi_{\mathrm{ich}}\left(\bar{C}\right):C\right]C^{-1}\right]$$

for notational simplicity. It is noted that the well-established relations $\partial_{C}J=\frac{1}{2}\;J\;C{}^{-1}$ and $\partial_{C}\bar{C}=J^{-\frac{2}{3}}\left[I-\frac{1}{3}\;C⊗C{}^{-1}\right]$ were employed here.

The corresponding hyperelastic part of the spatial Kirchhoff stresses, $\tau_{\mathrm{hyp}}=F\cdot S_{\mathrm{hyp}}\cdot F^{t}$, then follows as

(6) $$\tau_{\mathrm{hyp}}=J\;\partial_{J}\psi_{\mathrm{vol}}\left(J\right)\;I+\mathrm{dev}\;\left(2\;\bar{F}\cdot \partial_{\bar{C}}\psi_{\mathrm{ich}}\left(\bar{C}\right)\cdot \bar{F}{}^{t}\right)\;$$

with $\mathrm{dev}\left(•\right)=\left[•\right]-\frac{1}{3}\;\left[\left[•\right]:I\right]I$ being the deviator operator in spatial representation.

### 3.2. Extension of Finite Hyperelasticity to Finite Viscoelasticity

In view of a generalisation of the volumetrically-isochorically decoupled finite strain hyperelastic modelling framework outlined in Section 3.1 to finite viscoelasticity, C(t) is now assumed to be a function of time *t*. As a relation for the viscoelastic stress response in terms of Piola–Kirchhoff stresses S at time *t*, the form

(7) $$S\left(t\right)=S_{\mathrm{hyp}}\left(t\right)-J^{-\frac{2}{3}}\sum_{i=1}^{N}\mathrm{DEV}\left(Q_{i}\left(t\right)\right)$$

is considered and is in line with classic works such as [5,23], where $\mathrm{DEV}\left(•\right):=\left[•\right]-\frac{1}{3}\left[\left[•\right]:C\right]C^{-1}$. Here, $S_{\mathrm{hyp}}\left(t\right)$ denotes the hyperelastic stress contribution defined in (4) and $Q_{i}\left(t\right)$; i=1,2,⋯,N represent stress-type tensor-valued internal variables. This form of the stress extension leaves the volumetric part of the constitutive response unaffected by viscous effects. Relation (7) reflects a finite strain generalisation of a classic generalised relaxation model with *N* being the number of Maxwell elements, each consisting of a (linear) spring and a (linear) dashpot. Conceptually speaking, in a simplified and linear setting, each Maxwell element would be characterised by a given spring stiffness, say $E_{i}$, and corresponding dashpot viscosity, say $\eta_{i}$; cf., e.g., [5].

For the evolution of the stress-type internal variables, rate equations of the type

(8) $${\dot{Q}}_{i}\left(t\right)+\frac{1}{\tau_{i}}\;Q_{i}\left(t\right)=\frac{\gamma_{i}}{\tau_{i}}\;\mathrm{DEV}\;\left(2\;\partial_{\bar{C}}\psi_{\mathrm{ich}}\left(\bar{C}\left(t\right)\right)\right)\;\mathrm{with}\;\lim_{t\to -\infty }Q_{i}\left(t\right)=0\;,$$

are considered (cf. [5,23]), where $\tau_{i}$ are referred to as relaxation times and where $\gamma_{i}$ represent relative moduli subject to the restriction

(9) $$\sum_{i=1}^{N}\gamma_{i}=1-\gamma_{\infty }\;,$$

with $\gamma_{\infty }\in \left[0,1\right)$ determining the quasi-static (hyperelastic) limiting case of the material response. In a simplified and linear setting, as thoroughly introduced in [5], $\tau_{i}:=\eta_{i}/E_{i}>0$ and $\gamma_{i}=E_{i}/E_{0}\in \left[0,1\right]$ would be the parameters defining the properties of each Maxwell branch in the viscoelastic model. Here, $E_{0}=E_{\infty }+\sum_{i}E_{i}$ is the stiffness obtained in the (hyperelastic) limiting case reflected by infinitely high strain rates. In the context of the three-dimensional non-linear formulation at hand, this contribution is represented by the general Helmholtz free energy potential $\psi_{\mathrm{hyp}}$ that facilitates the incorporation of higher-order and even anisotropic constitutive relations.

The linearity of evolution Equation (8) facilitates the standard closed-form convolution representation

(10) $$Q_{i}\left(t\right)=\frac{\gamma_{i}}{\tau_{i}}\int_{-\infty }^{t}\exp\left(-\left(t-s\right)/\tau_{i}\right)\;\mathrm{DEV}\;\left(2\;\partial_{\bar{C}}\psi_{\mathrm{ich}}\left(\bar{C}\left(s\right)\right)\right)ds\;.$$

Combining (10) with (7) and integrating by parts yield an explicit expression for the stress response in terms of the Piola–Kirchhoff stress tensor of the form

(11) $$S\left(t\right)=J\;\partial_{J}\psi_{\mathrm{vol}}\left(J\right)\;C^{-1}\left(t\right)+J^{-\frac{2}{3}}\left(t\right)\int_{-\infty }^{t}g\left(t-s\right)\;\frac{d}{ds}\;\left(\mathrm{DEV}\;\left(2\;\partial_{\bar{C}}\psi_{\mathrm{ich}}\left(\bar{C}\left(s\right)\right)\right)\right)ds\;,$$

where the relaxation function

(12) $$g\left(t\right)=\gamma_{\infty }+\sum_{i=1}^{N}\gamma_{i}\;\exp\left(-t/\tau_{i}\right)$$

is incorporated.

The spatial counterpart of (11) is obtained by using $J^{-\frac{2}{3}}\;F\cdot \left[\mathrm{DEV}\left(•\right)\right]=\mathrm{dev}\left(\bar{F}\cdot \left(•\right)\cdot \bar{F}{}^{t}\right)$, so that the convolution representation of the Kirchhoff stresses reads

(13) $$\tau \left(t\right)=J\;\partial_{J}\psi_{\mathrm{vol}}\left(J\right)\;I+\int_{-\infty }^{t}g\left(t-s\right)\;\frac{d}{ds}\;\left(\mathrm{dev}\;\left(2\;\bar{F}\cdot \partial_{\bar{C}}\psi_{\mathrm{ich}}\left(\bar{C}\left(s\right)\right)\cdot \bar{F}{}^{t}\right)\right)ds\;.$$

For more detailed information on the derivation of well-established relations in the context of (undamaged) finite strain viscoelasticity based on a volumetric-isochoric split of the underlying Helmholtz free energy function, the reader is referred to classic monographs such as [5,24], amongst other works.

The numerical solution of the convolution integrals introduced above is based on a transformation towards a recurrence formula, enabling the computation of the constitutive response based on the standard internal variable procedure, thereby bypassing the need to store the entire strain history that is individual to each material point in view of the later finite element application; see Appendix A for details on the numerical update procedure.

### 3.3. Incorporation of Damage

We considered a gradient-regularised damage formulation in view of a mesh-objective solution of boundary value problems by using the finite element method, where the constitutive response can include damage-induced softening. To this end, an overall Helmholtz free energy potential ψ is introduced, composed of local and non-local energy contributions, $\psi_{\mathrm{loc}}$ and $\psi_{\mathrm{nloc}}$, in terms of $\psi =\psi_{\mathrm{loc}}+\psi_{\mathrm{nloc}}$.

The local Helmholtz free energy contribution, in extension of (3), takes the form

(14) $$\psi_{\mathrm{loc}}\left(C,\kappa \right)=f_{d}\left(\kappa \right)\;\psi_{\mathrm{vol}}\left(J\right)+f_{d}^{n_{\mathrm{iso}}}\left(\kappa \right)\;\psi_{\mathrm{ich}}\left(\bar{C}\right)\;,$$

where the volumetric and isochoric damage functions, $f_{d}\in \left(0,1\right]$ and $f_{d}^{n_{\mathrm{iso}}}\in \left(0,1\right]$, are evaluated based on a common internal damage variable κ, but allow for an adjustment of the volumetric-isochoric damage distribution via the exponent $n_{\mathrm{iso}}\in R$. The exponent $n_{\mathrm{iso}}$ is a material parameter that facilitates the modelling of a damage process that affects volumetric and isochoric contributions with different intensities.

The non-local energy contribution $\psi_{\mathrm{nloc}}$ enables the damage regularisation and consists of two contributions, namely a non-local gradient term $\psi_{\mathrm{nloc}}^{\mathrm{grad}}\left(\nabla_{\;X}\phi ;F\right)$ and a non-local penalty term $\psi_{\mathrm{nloc}}^{\mathrm{plty}}\left(\phi ,\kappa \right)$, so that $\psi_{\mathrm{nloc}}\left(F,\phi ,\nabla_{\;X}\phi ,\kappa \right)=\psi_{\mathrm{nloc}}^{\mathrm{grad}}\left(\nabla_{\;X}\phi ;F\right)+\psi_{\mathrm{nloc}}^{\mathrm{plty}}\left(\phi ,\kappa \right)$, with

(15) $$\begin{array}{cc} \psi_{\mathrm{nloc}}^{\mathrm{grad}}\left(\nabla_{\;X}\phi ;F\right) & =\frac{c_{d}}{2}\;\nabla_{\;X}\phi \cdot C{}^{-1}\cdot \nabla_{\;X}\phi =\frac{c_{d}}{2}\;\nabla_{\;x}\phi \cdot \nabla_{\;x}\phi \;, \end{array}$$

(16) $$\begin{array}{cc} \psi_{\mathrm{nloc}}^{\mathrm{plty}}\left(\phi ,\kappa \right) & =\frac{\beta_{d}}{2}\;{\left[\phi -\kappa \right]}^{2}\;. \end{array}$$

Here, ϕ is the non-local damage variable that is coupled to the local damage variable κ via the penalty parameter $\beta_{d}$. Moreover, $c_{d}$ represents a length scale-type regularisation parameter. With this particular choice of the non-local energy contributions, the Euler–Lagrange equations for the non-local damage field variable in spatial format take the form

(17) $$\begin{array}{cc} c_{d}\;\nabla_{\;x}\cdot \left[\nabla_{\;x}\phi \right]-\beta_{d}\;\left[\phi -\kappa \right] & =0\;\;\mathrm{in}\;B_{t}\;, \end{array}$$

(18) $$\begin{array}{cc} \nabla_{\;x}\phi \cdot n & =0\;\;\mathrm{on}\;\partial B_{t}^{y}=\partial B_{t}\;; \end{array}$$

see, e.g., [6] for more details on the associated relations, as well as on the numerical treatment within appropriately coupled finite element formulations.

The incorporation of the damage functions in the local free energy potential (14) induces Piola–Kirchhoff stresses of the form

(19) $$S_{\mathrm{hyp}}:=2\;\partial_{C}\psi_{\mathrm{loc}}\left(C,\kappa \right)=J\;f_{d}\left(\kappa \right)\;\partial_{J}\psi_{\mathrm{vol}}\left(J\right)\;C^{-1}+f_{d}^{n_{\mathrm{iso}}}\left(\kappa \right)\;{\bar{S}}_{\mathrm{hyp}}\;,$$

corresponding to spatial Kirchhoff stresses given by

(20) $$\tau_{\mathrm{hyp}}=J\;f_{d}\left(\kappa \right)\;\partial_{J}\psi_{\mathrm{vol}}\left(J\right)\;I+\mathrm{dev}\;\left(2\;f_{d}^{n_{\mathrm{iso}}}\;\bar{F}\cdot \partial_{\bar{C}}\psi_{\mathrm{ich}}\left(\bar{C}\right)\cdot \bar{F}{}^{t}\right)\;,$$

both of which are direct extensions of their purely hyperelastic counterparts introduced in (4) and (6).

The damage function affecting the isochoric part of the free energy function furthermore affects the evolution of viscous stress-type internal variables via

(21) $${\dot{Q}}_{i}\left(t\right)+\frac{1}{\tau_{i}}\;Q_{i}\left(t\right)=\frac{\gamma_{i}}{\tau_{i}}\;f_{d}^{n_{\mathrm{iso}}}\;\mathrm{DEV}\;\left(2\;\partial_{\bar{C}}\psi_{\mathrm{ich}}\left(\bar{C}\left(t\right)\right)\right)\;,$$

which basically reflects the damage-extended version of (8). This induces a convolution representation in terms of

(22) $$Q_{i}\left(t\right)=\frac{\gamma_{i}}{\tau_{i}}\int_{-\infty }^{t}\exp\left(-\left(t-s\right)/\tau_{i}\right)\;f_{d}^{n_{\mathrm{iso}}}\;\mathrm{DEV}\;\left(2\;\partial_{\bar{C}}\psi_{\mathrm{ich}}\left(\bar{C}\left(s\right)\right)\right)ds\;,$$

see (10) for the corresponding expression that is associated with an undamaged setting.

Finally, the Piola–Kirchhoff stresses that fully incorporate viscous and damage effects take the form

(23) $$S\left(t\right)=Jf_{d}\;\partial_{J}\psi_{\mathrm{vol}}\left(J\right)\;C^{-1}\left(t\right)+J^{-\frac{2}{3}}\left(t\right)\int_{-\infty }^{t}g\left(t-s\right)\;\frac{d}{ds}\;\left(f_{d}^{n_{\mathrm{iso}}}\;\mathrm{DEV}\;\left(2\;\partial_{\bar{C}}\psi_{\mathrm{ich}}\left(\bar{C}\left(s\right)\right)\right)\right)ds\;,$$

which allows for the accommodation of arbitrary free energy functions, in particular energy functions of higher order such as the Yeoh model that appropriately captures the Treloar-type behaviour of materials such as the particular elastomer considered in this work.

The numerical solution of the above-introduced convolution integrals is based on a transformation towards a recurrence formula, enabling the computation of the constitutive response based on the standard internal variable procedure, thereby bypassing the need to store the entire strain history that is individual to each material point in view of the later finite element application; see Appendix A for details on the numerical update procedure.

## 4. Parameter Identification

This section discusses the optimisation-based procedures employed for the identification of the constitutive parameters of the finite strain viscoelastic material model coupled with gradient-enhanced continuum damage as introduced in Section 3. Since the general framework can be applied to all kinds of energy functions, at first, considering the type of material and its behaviour, the chosen hyperelastic material model is briefly presented in Section 4.1. Next, the applied damage function $f_{d}$ is specified in Section 4.2. The main idea is to first carry out a parameter identification based on homogeneous states of deformation as described in Section 4.3. The obtained set of material parameters is then used as the initial value for the parameter identification based on inhomogeneous states of deformation, as elaborated in Section 4.4. The gradient terms of the gradient-enhanced damage framework are activated only in the case of inhomogeneous deformations.

### 4.1. Hyperelastic Material Model

Considering the nearly incompressible, non-linear elastic material behaviour (see for example the stress-strain-response in Figure 2b), the Yeoh hyperelastic material model was chosen [25,26]. In [27], Yeoh and Flemming argued that Rivlin’s theory [28], where the strain energy function depends on the first two invariants of the right Cauchy–Green tensor C, to be specific

(24) $$\begin{array}{c} \psi_{\mathrm{Riv}}=\sum_{i,j=1}^{\infty }C_{ij}{\left[I_{1}-3\right]}^{i}{\left[I_{2}-3\right]}^{j}\;, \end{array}$$

runs into difficulties with respect to the identification of the material parameters since the contributions cannot be perfectly determined separately in the experiments. Here, $C_{ij}$ are the corresponding material parameters, and $I_{1}$ and $I_{2}$ are the first and second principal invariants related to the isochoric right Cauchy–Green tensor, respectively. Yeoh neglected the contribution of the second invariant assuming that no serious error would arise since the contribution would be sufficiently small with respect to the impact of the first invariant. In this work, however, the Yeoh energy potential

(25) $$\begin{array}{c} \psi_{\mathrm{ich}}=\sum_{i=1}^{3}C_{i}{\left[\mathrm{tr}\left(\bar{C}\right)-3\right]}^{i}\;, \end{array}$$

serves as the isochoric contribution to overall energy potential, where $C_{i}$ denote the underlying material parameters. This phenomenological material model was developed for rubber elasticity and is applied in this work as a first choice with respect to the mentioned material behaviour. The volumetric energy contribution $\psi_{\mathrm{vol}}$ is chosen in a standard manner as

(26) $$\begin{array}{c} \psi_{\mathrm{vol}}=\frac{1}{2}\;K\left[\frac{1}{2}\;\left[J^{2}-1\right]-\ln\left(J\right)\right]\;, \end{array}$$

where *K* denotes the bulk modulus of the material. The related energy contributions $\psi_{\mathrm{ich}}$ and $\psi_{\mathrm{vol}}$ can, however, be straightforwardly replaced within the general constitutive framework elaborated in this work in order to account for different particular material characteristics.

### 4.2. Damage Function

The damage function mentioned in Section 3 was chosen to be of an exponential type, following [6,29]. Thus, a damage initiation threshold, as well as a damage saturation rate can be used in the function

(27) $$\begin{array}{c} f_{d}\left(\kappa \right)=1-d=\exp\left(-\eta_{d}\langle \kappa -\kappa_{d}\rangle \right)\;, \end{array}$$

fulfilling the requirements for the damage function to be restricted to $f_{d}:R^{+}\to \left(0,1\right]$. Here, *d* denotes the classic damage variable with d=0 indicating zero damage and d=1 for 100% damage. The variable κ represents a related internal damage variable, while $\kappa_{d}\;>\;0$ is the damage threshold parameter, and $\eta_{d}$ introduces the damage saturation parameter. In addition, 〈•〉:=max{0,•} represents the Macaulay brackets. Consequently, damage is obtained in the material if the local damage variable exceeds the damage threshold. The damage function for the isochoric energy contribution matches this chosen function except for the exponent $n_{\mathrm{iso}}$. Thus, a relation between both functions is still given and only adjusted via this material parameter. The evolution of the internal damage variable κ is based on the associated form

(28) $$\begin{array}{c} \dot{\kappa }=\lambda \;\frac{\partial \Phi_{d}\left(F,\phi ,\nabla_{\;X}\phi ,\kappa \right)}{\partial q}\;, \end{array}$$

where λ denotes a proper Lagrange multiplier, $\Phi_{d}\left(F,\phi ,\nabla_{\;X}\phi ,\kappa \right)=q\left(F,\phi ,\nabla_{\;X}\phi ,\kappa \right)-\kappa \leq 0$ represents the damage condition, and $q=-\;\partial [\psi_{\mathrm{loc}}+\psi_{\mathrm{nloc}}]/\partial d$ is the energy release rate. Hence, apart from the balance of linear momentum, the Euler–Lagrange equation governing the non-local damage variable needs to be solved simultaneously. For further information regarding this regularised damage model and its implementation, see Ostwald et al. [6]. The implementation was performed in a user material subroutine (UMAT) in Abaqus following [6].

### 4.3. Parameter Identification Based on Homogeneous States of Deformation

In the context of parameter identification procedures, it is advantageous to compute the Piola stress tensor as a quantity, facilitating the comparison of experimental and simulated material response; cf. Section 2. The Piola stress tensor P is related to the Piola–Kirchhoff stress tensor S and the Kirchhoff stress tensor τ via

(29) $$P=\tau \cdot F^{t}=F\cdot S\;,$$

where the sensitivity of the Piola stress with respect to the deformation gradient is denoted as A, in particular,

(30) $$A=\frac{\partial P}{\partial F}=\frac{\partial^{2}\psi_{\mathrm{loc}}}{\partial F⊗\partial F}\;,$$

or, expressed in index notation,

(31) $$A_{aAbB}=\frac{\partial^{2}\psi_{\mathrm{loc}}}{\partial F_{aA}\;\partial F_{bB}}\;.$$

Moreover, A relates to the spatial elasticity tensor e via

(32) $$A_{aBcD}=F_{Bb}^{-1}\left[e_{abcd}+\tau_{ac}\delta_{bd}\right]F_{Dd}^{-1}\;.$$

It is noted that the tangent operator, as typically required within implicit finite element formulations, respectively so-called constitutive drivers, is based on A=dP/dF.

The Yeoh-type hyperelastic energy function was combined with the constitutive viscoelasticity model, presented in Section 3, including two Maxwell elements. Apart from the Yeoh material parameters $C_{1}$, $C_{2}$ and $C_{3}$, the viscoelastic parameters, $\gamma_{1}$ and $\gamma_{2}$, denoting the relation of the stiffness of each Maxwell element with respect to the pure elastic Young’s modulus, and $\tau_{1}$, $\tau_{2}$, representing the ratio of the viscosity to the stiffness in each Maxwell element, need to be identified. Regarding the parameters, it has to be mentioned that the material was assumed to be nearly incompressible, and thus, Poisson’s ratio was fixed to ν=0.49. Thus, seven material parameters needed to be identified.

Since these seven material parameters could be identified via experiments of homogeneous deformation states, strain- and stress-driven constitutive drivers were implemented in MATLAB instead of full finite element (FE) simulations in order to reduce the computational cost within each iteration of the parameter identification. The pseudo-codes of both types of constitutive drivers are depicted in Algorithms 1 and 2. The fminsearch-algorithm in MATLAB was used for the parameter identification, minimising the goal function with respect to the difference in the simulated and experimental reaction force over all load steps, i.e.,

(33) $$\begin{array}{cc} f_{\mathrm{obj}}=\; & w^{T}\;\sum_{t}w_{t}^{T}\;{\left|P_{t}^{T,\exp}-P_{t}^{T,\mathrm{sim}}\left(\kappa \right)\right|}^{2}+w^{R}\;\sum_{t}w_{t}^{R}\;{\left|P_{t}^{R,\exp}-P_{t}^{R,\mathrm{sim}}\left(\kappa \right)\right|}^{2}\\ \; & +w^{C}\;\sum_{t}w_{t}^{C}\;{\left|u_{t}^{C,\exp}-u_{t}^{C,\mathrm{sim}}\left(\kappa \right)\right|}^{2}\;, \end{array}$$

where $w^{T}$, $w^{R}$, and $w^{C}$ denote the weighting factors for the tensile, creep, and relaxation test, respectively. The additional weighting factors $w_{t}^{T}$, $w_{t}^{R}$, and $w_{t}^{C}$ were introduced to emphasise specific time steps *t* of each experiment.

In the following, the viscoelastic material parameters are identified via the three tests based on homogeneous deformation states, presented in Section 2.1.

**Figure 6:**
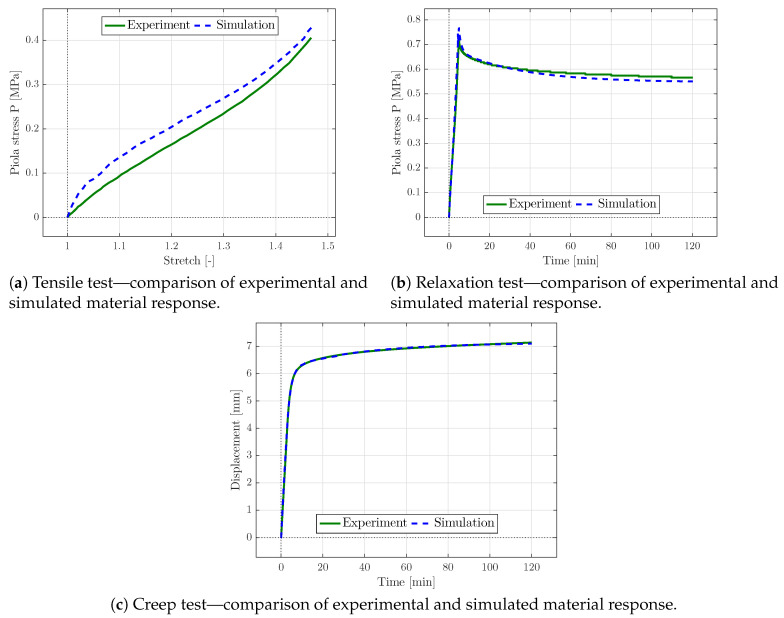
Comparison of experimental and simulation results of the (**a**) tensile, (**b**) relaxation, and (**c**) creep tests for uni-axial stress states, using the optimised set of material parameters; cf. Table 1.

If the parameters of the Yeoh-model were solely fitted with respect to the tensile test, the simulated material response of the identified parameters matched the experimental data perfectly. Next, the relaxation parameters were obtained with respect to the relaxation and creep test, though the material behaviour highly depended on the previously identified Yeoh parameters. Thus, the experimental relaxation and creep response could not be sufficiently matched via optimisation of the relaxation parameters only. Consequently, all of the material parameters were identified simultaneously for all three different experiments in order to obtain the best parameter set reflecting the complete material behaviour. The comparison of the simulated and experimental material behaviour is shown in Figure 6 for the optimised parameter set. The corresponding parameter set is presented in Table 1.

In the graphs shown in Figure 6, the experimental results of the relaxation and creep tests are nearly perfectly matched, and only slight differences in the diagrams can be seen at certain points. The simulated material response of the tensile test overestimated the experimental curve, but captured the general trend. The tensile test could be captured better by increasing the associated weight within the objective function (33) at the cost of the accuracy with which creep and relaxation tests were captured. The focus here was set on the rate-dependent constitutive characteristics that were captured with high accuracy. Though beyond the scope of this work, a precise capturing of tensile, creep, and relaxation test at the same time could be achieved by introducing higher order energy functions and a larger number of independent Maxwell branches within the constitutive framework provided. The accompanying increase in parameter identification complexity due to a significantly increased number of constitutive parameters could then be dealt with using parameter correlation matrices.

**Algorithm 1** Strain-driven constitutive driver (relaxation)—uni-axial stress state; see also Appendix A.
  1:initialize internal variables ${\tilde{S}}_{\mathrm{hyp}}^{n},\;H_{n}^{\left(i\right)},\forall \;i=1,\cdots ,N$.  2:get material parameters $C_{1},\;C_{2},\;C_{3},\;\gamma_{1},\;\tau_{1},\cdots ,\;\gamma_{i},\;\tau_{i}$.  3:initialize $P_{n+1}$.  4:**for** every time step $t_{n}$ **do**  5:      given: the stretch $\lambda_{11}$.  6:      **while** $∥{\hat{P}}_{n+1}∥<tol$ **do**  7:            compute ${}^{\left(k\right)}F_{n+1}$, ${}^{\left(k\right)}J_{n+1}$, ${}^{\left(k\right)}C_{n+1}$, ${}^{\left(k\right)}{\hat{F}}_{n+1}$, ${}^{\left(k\right)}{\hat{C}}_{n+1}$.  8:            compute initial elastic stress Kirchhoff tensor $\tau_{n+1}^{\mathrm{hyp}}$.  9:            compute algorithmic internal variables ${\tilde{S}}_{\mathrm{hyp}}^{n+1}$, $H_{n+1}^{\left(i\right)}$.10:            compute ${}^{\left(k\right)}S_{n+1}$ according to (A6).11:            compute the spatial tangent moduli ${}^{\left(k\right)}{\hat{e}}_{n+1}$.12:            compute the Piola–Kirchhoff stress tensor ${}^{\left(k\right)}P_{n+1}$.13:            compute the tangent operator ${}^{\left(k\right)}A_{n+1}$.14:            partition the stress tensor and the deformation gradient:15:            ${}^{\left(k\right)}P_{n+1}={}^{\left(k\right)}P_{11}\;e_{11}⊗e_{11}+{}^{\left(k\right)}{\hat{P}}_{n+1}$16:            ${}^{\left(k\right)}F_{n+1}={}^{\left(k\right)}F_{11}\;e_{11}⊗e_{11}+{}^{\left(k\right)}{\hat{F}}_{n+1}$.17:            partition the tangent operator:18:            ${}^{\left(k\right)}{\hat{A}}_{n+1}=\frac{{}^{\left(k\right)}d{\hat{P}}_{n+1}}{{}^{\left(k\right)}d{\hat{F}}_{n+1}}$.19:            update the transverse deformation gradient:20:            ${}^{\left(k+1\right)}{\hat{F}}_{n+1}\leftarrow {}^{\left(k\right)}{\hat{F}}_{n+1}-{}^{\left(k\right)}{\hat{A}}_{n+1}:{}^{\left(k\right)}{\hat{P}}_{n+1}$.21:      **end while**22:      assemble the deformation gradient and the stress tensor $F_{n+1},\;P_{n+1}$23:      update internal variables $\left\{{\tilde{S}}_{\mathrm{hyp}}^{n+1},\;H_{n+1}^{\left(i\right)}\right\}\leftarrow \left\{{\tilde{S}}_{\mathrm{hyp}}^{n},\;H_{n}^{\left(i\right)}\right\}$.24:
**end for**



Considering the curve of the tensile test, especially the values and the signs of the Yeoh parameters are important, since the first parameter $C_{1}$ weights the linear behaviour of the stress strain-response, whereas $C_{2}$ scales the quadratic term of the isochoric energy contribution resulting in the decreasing slope. The third parameter $C_{3}$ corresponds to the third-order polynomial contribution of the energy function and thus yields the increasing slope at the end of the loading path. Thus, different values for the Yeoh parameters would probably fit the tensile response way better; the dependence on the relaxation parameters, however, would result in a worse fit of the relaxation and creep behaviour. Higher order contributions to the Helmholtz free energy, in particular the isochoric contribution, would therefore provide a potential in order to capture the tensile test response better.

**Algorithm 2** Stress-driven constitutive driver (creep)—uni-axial stress state; see also Appendix A.
  1:initialize internal variables ${\tilde{S}}_{\mathrm{hyp}}^{n},\;H_{n}^{\left(i\right)},\forall \;i=1,\cdots ,N$.  2:get material parameters $C_{1},\;C_{2},\;C_{3},\;\gamma_{1},\;\tau_{1},\cdots ,\;\gamma_{i},\;\tau_{i}$.  3:initialize $F_{n+1}$.  4:**for** every time step $t_{n}$ **do**  5:        given: Piola–Kirchhoff stress tensor $P^{\exp}$.  6:        **while** $∥P-P^{\exp}∥<tol$ **do**  7:              compute ${}^{\left(k\right)}F_{n+1}$, ${}^{\left(k\right)}J_{n+1}$, ${}^{\left(k\right)}C_{n+1}$, ${}^{\left(k\right)}{\hat{F}}_{n+1}$, ${}^{\left(k\right)}{\hat{C}}_{n+1}$.  8:              compute initial elastic Kirchhoff stress tensor $\tau_{n+1}^{\mathrm{hyp}}$.  9:              compute algorithmic internal variables ${\tilde{S}}_{\mathrm{hyp}}^{n+1},H_{n+1}^{\left(i\right)}$.10:              compute ${}^{\left(k\right)}S_{n+1}$ according to (A6).11:              compute the spatial tangent moduli ${}^{\left(k\right)}{\hat{e}}_{n+1}$.12:              compute Piola–Kirchhoff stress tensor ${}^{\left(k\right)}P_{n+1}$.13:              compute tangent operator ${}^{\left(k\right)}A_{n+1}$.14:              compute ${}^{\left(k\right)}\Delta P_{n+1}={}^{\left(k\right)}P_{n+1}-{}^{\left(k\right)}P_{n+1}^{\exp}$.15:              update the deformation gradient16:              ${}^{\left(k+1\right)}F_{n+1}\leftarrow {}^{\left(k\right)}F_{n+1}-{}^{\left(k\right)}A_{n+1}:{}^{\left(k\right)}\Delta P_{n+1}$17:        **end while**18:        update internal variables $\left\{{\tilde{S}}_{\mathrm{hyp}}^{n+1},\;H_{n+1}^{\left(i\right)}\right\}\leftarrow \left\{{\tilde{S}}_{\mathrm{hyp}}^{n},\;H_{n}^{\left(i\right)}\right\}$19:
**end for**



The identified viscoelastic material parameters were used as fixed values for the subsequent optimisation of the damage-related parameters.

### 4.4. Parameter Identification Based on Inhomogeneous States of Deformation

In the following, the previously identified Yeoh and relaxation parameters are used to identify the damage-related material parameters $\eta_{d}$, $\kappa_{d}$, $n_{\mathrm{iso}}$, and $c_{d}$ via the tensile test with inhomogeneous deformation states presented in Section 2.2. In contrast to the identification of the viscoelastic parameters, the constitutive driver is not sufficient enough to capture the material behaviour of the gradient-enhanced damage model. Thus, a finite element (FE) formulation is required. Therefore, the material model was implemented into a UMAT in Abaqus, as already mentioned in Section 4.2.

In addition, a parameter identification tool was implemented in Python, using the Nelder–Mead-Simplex algorithm of the scipy-package. In contrast to the calibration of the Yeoh and relaxation parameters, it did not suffice to solely consider the difference in the Piola stress $P_{11}$ or the (clamping) displacement $u_{1}$, depending on the experiment, for the goal function $f_{\mathrm{obj}}$ of the optimisation. Since the gradient terms—capturing the mesh-objectivity of damage—of the regularised damage framework are activated by inhomogeneous deformation states, the difference in the displacement field between the simulated and experimental material response needs to be added to the goal function apart from the difference in the reaction force

(34) $$\begin{array}{c} f_{\mathrm{obj}}=\sum_{t}\sum_{i=1}^{n_{\mathrm{np}}}w_{u}\;{∥u_{ti}^{\exp}-u_{ti}^{\mathrm{sim}}\left(\kappa \right)∥}^{2}+\sum_{t}w_{F}\;{\left|F_{t}^{\exp}-F_{t}^{\mathrm{sim}}\left(\kappa \right)\right|}^{2}\;, \end{array}$$

where $n_{\mathrm{np}}$ denotes the number of node points considered, $w_{u}$ the weighting factor for the displacement contribution, and $w_{F}$ the weighting factor for the impact of the reaction force. The number of node points was the element nodes on the surface of the specimen in the FE simulations. The experimental data was interpolated onto those node points via a 2D-interpolation scheme prior to the parameter identification following Kleuter [13].

**Figure 7:**
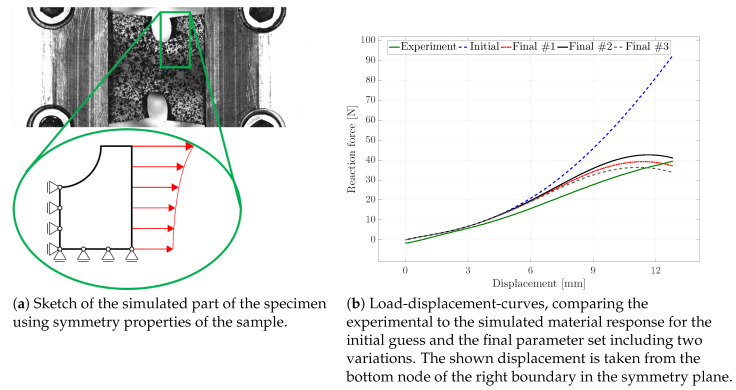
(**a**) Sketch of the chosen segment of the specimen with the applied boundary conditions. (**b**) Comparison of the load-displacement curves of the experiment with the results of the initial parameter set, the optimised Parameter Sets #1 ($n_{\mathrm{iso}}\;=\;1.0$), #2 ($n_{\mathrm{iso}}\;=\;0.9$), and #3 ($n_{\mathrm{iso}}\;=\;1.1$).

For the purpose of reducing the computational cost of the FE simulation within each iteration of the parameter identification, the symmetry properties of the sample were used; cf. Figure 7a. Furthermore, considering the material properties of the soft polymer, only the segment shown in the figure was used to exclude boundary effects of the clamping jaws. In order to still use the experimental boundary conditions in the simulations, the experimentally measured displacements, taken via the CCD camera system, were applied to the right boundary of the specified segment. As can be seen from the photos of the specimen during the experiment (cf. Figure 5) and from the sketch of the segment, the displacements were not uniform over the boundary of the chosen segment of the sample.

**Figure 8:**
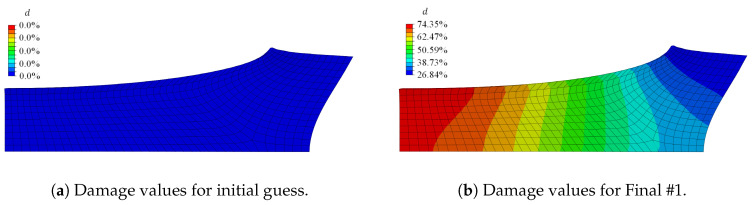
Comparison of the contour plot of the damage value *d* at the final load step for (**a**) the initial parameter set and (**b**) the optimised Parameter Set #1.

The initial guess for the damage-related model parameters was $\eta_{d}\;=\;0.002$, $\kappa_{d}\;=\;0.4$, $n_{\mathrm{iso}}\;=\;1.0$, and $c_{d}\;=\;0.04$ and resulted in no damage initiation in the material; cf. Figure 8a. In Figure 7b, the comparison of the load-displacement curves is presented. Since the stress-strain path of the homogeneous tensile test already overestimated the experimental curve, the simulated response for the tensile test with inhomogeneous deformation states lied above the experimental result as well. Furthermore, considering the large total stretch values present in combination with the identified Yeoh material parameters, the increasing response for the reaction force was triggered by the parameters $C_{2}$ and $C_{3}$ weighting the hyperelastic energy contributions of second and third order.

**Figure 9:**
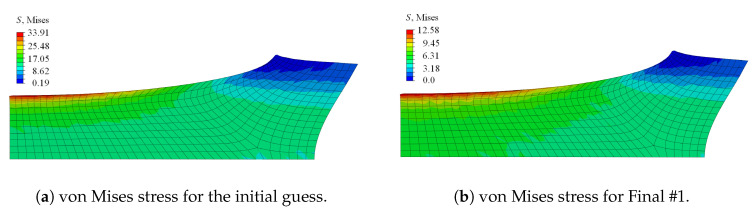
Comparison of the contour plot of the von Mises stress at the final load step for (**a**) the initial parameter set and (**b**) the optimised Parameter Set #1.

Since parameter $n_{\mathrm{iso}}$ strongly influences the simulated deformation behaviour of the specimen in each iteration, $n_{\mathrm{iso}}$ was fixed to 1.0 in a first step, thereby neglecting a different damage contribution of the volumetric and isochoric part. The corresponding optimised parameter set, denoted as Final #1, $\eta_{d}\;=\;0.202531$, $\kappa_{d}\;=\;0.15185$, and $c_{d}\;=\;0.52593$, was rounded to five decimal places. Considering the load-displacement curves, the response of the final set lied closer to the experimental behaviour than the initial guess; see Figure 7b. The stress distribution of the initial guess and the final set was comparable; the magnitude of the final set, however, was less than half of the stress of the initial set; cf. Figure 9. The von Mises stress distribution within the specimen—with the maximum stress value obtained at the upper surface, corresponding to the region dominated by the initially circular notch—was in line with observations made by, e.g., Kleuter [13].

The contour plot of the damage value *d* in Figure 8 compares the damage value obtained for the initial parameter set with the damage value obtained for the Final #1 set. Initially, no damage was initiated, and with the Final #1 set, a damage value of nearly 75% occurred in the symmetry plane of the loading direction.

**Figure 10:**
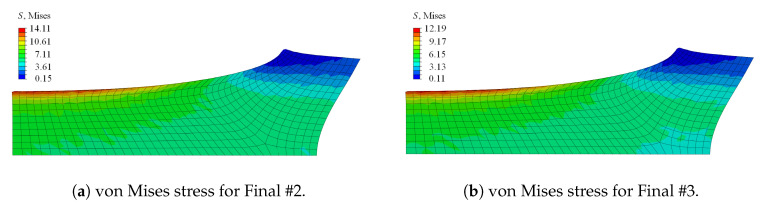
Comparison of the contour plot of the von Mises stress at the final load step for (**a**) the optimised Parameter Set #2 with $n_{\mathrm{iso}}\;=\;0.9$ and (**b**) the optimised Parameter Set #3 with $n_{\mathrm{iso}}\;=\;1.1$.

In Figure 7b and Figure 10, the influence of the different damage functions for the volumetric and isochoric contributions is visible. The set Final #2 included $n_{\mathrm{iso}}\;=\;0.9$, and Final #3 considered $n_{\mathrm{iso}}\;=\;1.1$. A higher value for $n_{\mathrm{iso}}$ flattens the load-displacement curve, while a lower value provides an increased path. Another important impact of the parameter is indicated in Figure 10. Apart from the difference in the stress magnitude, the deformation of the sample was already different for slight changes in the parameter with regard to the necking of the sample at the symmetry plane of the loading direction.

**Figure 11:**
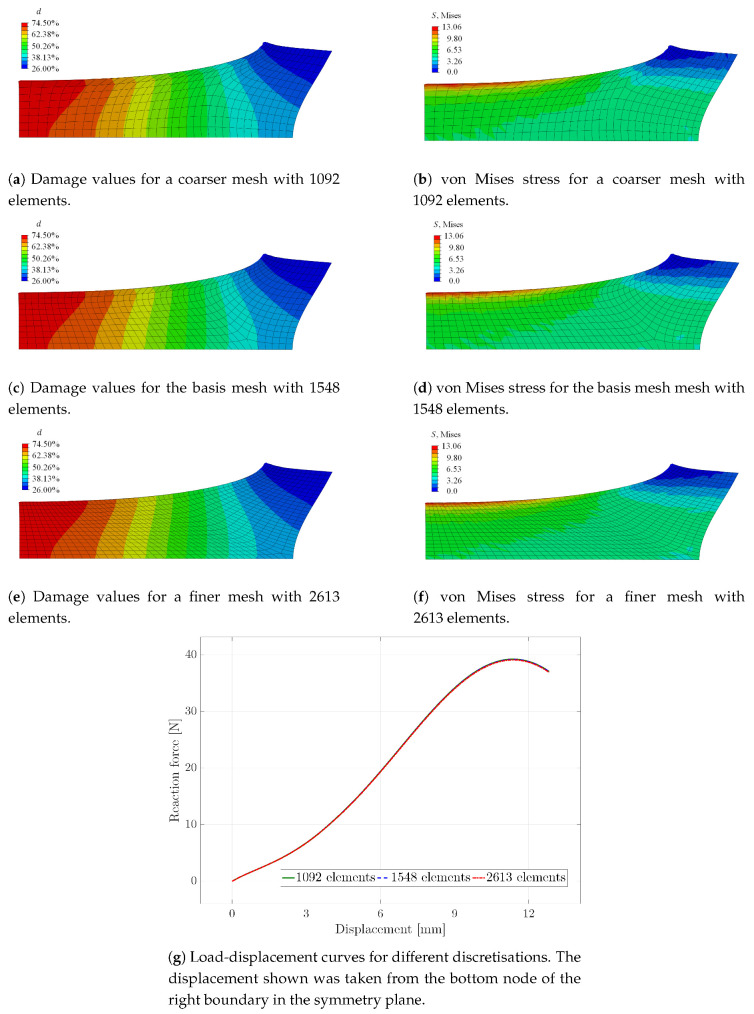
Contour plots of the damage function values and the von Mises stress, as well as load-displacement curves regarding the parameter set Final #1 for different discretisations.

The advantage of the regularised damage framework is the mesh objectivity of the simulated material response. To demonstrate this feature, the boundary value problem was calculated with three different meshes for the optimised parameter set. In addition to the mesh containing 1548 elements, used for the results in Figure 7, Figure 8, Figure 9 and Figure 10, a coarser mesh with 1092 and a finer mesh with 2613 elements were used in order to analyse the mesh sensitivity of the results. As can be seen in Figure 11, only marginal differences in the contour plots of the damage function values and the von Mises stress, as well as the load-displacement curves are visible. Thus, the gradient-enhanced damage model is working properly. In contrast, it is noted that the local damage model diverged at different load steps, depending on the mesh discretisation. In the case of the fine mesh, the solution diverged at a displacement of 3.2mm, for the basis mesh at 3.375mm and for the coarse mesh at 3.415mm. The displacement was taken from the bottom node of the right boundary in the symmetry plane.

## 5. Conclusions

In this work, a parameter identification framework for gradient-enhanced damage in rate-dependent materials under finite strains was introduced. In order to identify the set of over 12 material parameters, an efficient scheme for the process was carried out. At first, the basic constitutive model parameters, namely elastic parameters, were calibrated with respect to experiments displaying homogeneous states of deformation. Considering the deformation states, the computational cost of the identification was further reduced by using constitutive drivers for the simulations of all three required types of experiments within each iteration of the calibration process. In the next step, the already identified parameters were fixed during the identification of the damage-related material parameters.

For the purpose of developing a general framework for the calibration of rate-dependent materials coupled to damage, the constitutive viscoelasticity model by Simo and Hughes [5] was coupled to the gradient-enhanced damage formulation. Thus, the advantages of both models were combined, enabling different types of hyperelastic energy formulations—considering the energy-independent viscoelastic derivations by Simo and Hughes [5]—as well as mesh-independent results with respect to the damage evolution due to the damage regularisation framework by Ostwald et al. [6].

To investigate whether the framework works even for very large deformations, a self-diagnostic elastomer incorporating the mechanophore units within the polymeric microstructure was chosen as an application. With regard to the material behaviour of the soft polymer, the hyperelastic model by Yeoh and Fleming [27] was applied to the viscoelastic damage model.

The results of the parameter identification in Section 4 showed that the general framework of the implemented material model, as well as the parameter identification tool works properly. Even though the chosen self-diagnostic elastomer was only a first attempt for the application of the framework, the experimental results were already basically matched; cf. Figure 6 and Figure 7b. Since the simulations for the experiments with inhomogeneous deformation states were conducted before the sample started to tear in the necking region, the damage distribution with the optimised parameter set—showing damage evolution of nearly 75% in the symmetry plane of the loading direction, i.e., the necking region—reflected such a material behaviour.

Nevertheless, further improvements could be achieved for example by using different weighting factors for the contributions of the three different experiments with homogeneous deformation states to the goal function of the optimisation in order to improve the calibration of the parameters with respect to the tensile test. However, after all, the largest improvement could be achieved by a refinement of the experiments. The material properties led to difficulties in the sample manufacturing—such as uniformly cutting the shape—and in the clamping of the specimen, since the clamping jaws already generated deformations in the soft polymer. An improvement would be if the samples were directly moulded or if clamping were conducted with a very accurate torque spanner. Furthermore, experimental data based on different strain rates could be included in the identification framework.

In addition, in the next step, the load path for the inhomogeneous experiments could be changed to cyclic loading, improving the damage characterisation, though increasing the computational cost within each iteration of the parameter identification procedure. Nevertheless, further experiments including unloading should be included in the calibration process to enhance the accuracy. Until now, only damage activated prior rupture has been taken into account. As a long-term goal, the current framework should be combined with a rupture model to improve the overall prediction of the material behaviour.

After all, the general parameter identification framework provides an efficient scheme for the calibration of gradient-enhanced damage in rate-dependent material models under finite strains. In the future, an interesting extension of the parameter identification framework is the incorporation of the self-diagnostic properties of the elastomer in terms of additional field data that is considered within the objective function.

## Figures and Tables

**Table 1:** Optimised set of material parameters for the Yeoh material model, as well as the relaxation parameters for the two Maxwell elements.

Parameter	$C_{1}$	$C_{2}$	$C_{3}$	$\gamma_{1}$	$\gamma_{2}$	$\tau_{1}$	$\tau_{2}$
**Value**	0.19550588	0.11198637	0.00685930	0.12862148	0.36026686	1879.5892	68.729741

## Appendix A. Numerical Time-Integration of the Local Constitutive Relations

In order to transform the convolution integral expression obtained in Section 3.2 to a standard recurrence formula, we adopt the following internal algorithmic variables,

(A1) $$H^{\left(i\right)}\left(t\right):=\int_{-\infty }^{t}\exp\left[-\left(t-s\right)/\tau_{i}\right]\;\frac{d}{ds}\;\left(f_{d}^{n_{\mathrm{iso}}}\left(\kappa \right)\mathrm{DEV}\left(2\;\partial_{\bar{C}}\psi_{\mathrm{ich}}\left(\bar{C}\left(s\right)\right)\right)\right)ds\;,$$

where $H^{\left(i\right)}\left(t\right),i=1,2,\ldots ,N$, i.e., we have one stress-type tensor-valued internal variable $H^{\left(i\right)}$ for each Maxwell element considered in the generalised relaxation model; cf. Simo and Hughes [5].

Using the semigroup property exp([t+Δt]/a)=exp(Δt/a)exp(t/a) and the property of the additivity of the integral over the interval of integration, we obtain the recurrence relation

(A2) $$\begin{array}{cc} H^{\left(i\right)}\left(t_{n+1}\right) & =\exp\left[-\Delta t_{n}/\tau_{i}\right]\;H^{\left(i\right)}\left(t_{n}\right)\\ & +\int_{t_{n}}^{t_{n+1}}\exp\left[-\left(t_{n+1}-s\right)/\tau_{i}\right]\;\frac{d}{ds}\;\left(f_{d}^{n_{\mathrm{iso}}}\left(\kappa \right)\mathrm{DEV}\left(2\;\partial_{\bar{C}}\psi_{\mathrm{ich}}\left(\bar{C}\left(s\right)\right)\right)\right)ds\;. \end{array}$$

Finally, the integral (A2) is approximated using the midpoint rule, so that

(A3) $$H_{n+1}^{\left(i\right)}=\exp\left(-\Delta t/\tau_{i}\right)\;H_{n}^{\left(i\right)}+f_{d}^{n_{\mathrm{iso}}}\left(\kappa \right)\;\exp\left(-\Delta t/2\;\tau_{i}\right)({\tilde{S}}_{\mathrm{hyp}}^{n+1}-{\tilde{S}}_{\mathrm{hyp}}^{n})\;,$$

using the abbreviations

(A4) $${\tilde{S}}_{\mathrm{hyp}}^{n+1}:={\mathrm{DEV}}_{n+1}\left(2\;\partial_{\bar{C}}\psi_{\mathrm{ich}}\left({\bar{C}}_{n+1}\right)\right)\;\mathrm{and}\;{\tilde{S}}_{\mathrm{hyp}}^{n}:={\mathrm{DEV}}_{n}\left(2\;\partial_{\bar{C}}\psi_{\mathrm{ich}}\left({\bar{C}}_{n}\right)\right)\;,$$

where ${\mathrm{DEV}}_{n}$ and ${\mathrm{DEV}}_{n+1}$ are computed with respect to $C_{n}$ and $C_{n+1}$, respectively, consistent with

(A5) $${\mathrm{DEV}}_{n+1}\left(•\right)=\left[•\right]-\frac{1}{3}\left[\left[•\right]:C_{n+1}\right]C_{n+1}^{-1}\;.$$

The algorithmic approximation of the Piola–Kirchhoff stress tensor at time $t_{n+1}$ then takes the form

(A6) $$\begin{array}{cc} S_{n+1} & =J_{n+1}\;f_{d}\left(\kappa_{n+1}\right)\;\partial_{J}\psi_{\mathrm{vol}}\left(J_{n+1}\right)\;C_{n+1}^{-1}\\ \; & +\gamma_{\infty }\;f_{d}^{n_{\mathrm{iso}}}\left(\kappa_{n+1}\right)\;{\tilde{S}}_{\mathrm{hyp}}^{n+1}+\sum_{i=1}^{N}\gamma_{i}\;J_{n+1}^{-\frac{2}{3}}\;{\mathrm{DEV}}_{n+1}\left(H_{n+1}^{\left(i\right)}\right) \end{array}$$

with ${\tilde{S}}_{\mathrm{hyp}}^{n+1}$ representing relation (A4) evaluated at the current time $t_{n+1}$ and with $\kappa_{n+1}$ the (updated) internal variable representing the evolution of damage within the material. For details on the straightforward time-integration of κ via a standard Euler backward scheme, the reader may refer to, e.g., [6,29]. The spatial counterpart of (A6), i.e., the current approximation of the Kirchhoff stress tensor, is then computed using a standard push-forward operation in terms of

(A7) $$\tau_{n+1}=F_{n+1}\cdot S_{n+1}\cdot F{}_{n+1}^{t}\;.$$
